# Effect of asymmetry on frustrated crystallization

**DOI:** 10.1038/s41598-026-53474-8

**Published:** 2026-06-04

**Authors:** Carina Karner, Emanuela Bianchi

**Affiliations:** Institut für Theoretische Physik, TU Wien, Wiedner Hauptstraße 8-10, A-1040 Wien, Austria

**Keywords:** Chemistry, Materials science

## Abstract

Partial bonding in colloidal systems is often regarded as an obstacle to order, as it introduces geometric frustration. Yet, frustration can instead serve as a powerful design principle for controlling the formation of porous crystalline monolayers. By systematically varying the asymmetry in patch placement on anisotropic rhombic platelets, we investigate how partial bonding and geometric frustration can be tuned to direct polymorph selection, modulate porosity, and optimize crystal yield. This reveals an alternative route to crystallinity in patchy colloids – one that relies not on maximizing bonding, but on purposefully limiting it. Our findings establish a new strategy for programming functionality in colloidal materials, with relevance for the rational design of supramolecular architectures, DNA-based nanomaterials, and stimuli-responsive assemblies.

## Introduction

Patchy colloidal particles, whose interactions are localized to specific surface regions, have become increasingly programmable due to advances in synthesis and surface functionalization, enabling precise control over size, shape, and interaction specificity ^1–5^. Experimental realizations include DNA-coated colloidal molecules ^6^, functionalized DNA origami ^7,8^, and micron-scale spheres with patterned surface chemistry ^9^. By tuning patch type, number and geometry, these building blocks can be programmed to self-assemble into a variety of target structures, including colloidal diamond ^10^, Kagome lattice ^11^, and programmable 3D and 2D DNA-origami arrays ^12,13^.

This programmability has led to the emergence of inverse design strategies to identify the optimal patch arrangement/specificity for a given target structure, whether crystalline ^14–18^ or even quasicrystalline ^19,20^. The implicit assumption behind these efforts is that every patch should be involved in a single, well-defined bond with a neighboring patch, i.e., fully bonded configurations are generally considered a necessary condition for achieving long-range order in self-assembly.

This paradigm raises a fundamental question: what happens when particles are deliberately prevented from fully bonding? Partial bonding is often associated with geometric frustration, which tends to suppress long-range order and promote disordered aggregates. This expectation is rooted in the idea that if not all interaction sites can be simultaneously satisfied, the system faces conflicting local constraints. Moreover, when bonding is incomplete, the increased number of accessible local motifs typically leads to higher configurational entropy, which may hinder the formation of a uniform lattice. Indeed, recent inverse design strategies have begun targeting amorphous structures by encoding frustration directly into the building block design ^21^.

Frustration is nonetheless emerging as a powerful tool for steering assembly toward specific crystalline morphologies, challenging the conventional wisdom that frustration is inherently detrimental to order. For instance, geometrical frustration arising from mismatched particle geometries can promote the formation of low-dimensional structures such as crystalline fibers ^22^. In another example, the self-assembly of nanoparticles into non-Euclidean crystals has been shown to depend critically on the interplay between shape-induced frustration and competing attractive and repulsive forces ^23^. Likewise, subtle modifications in the shape and interaction zone of colloidal molecules can significantly influence the assembly from differently ordered monolayers to chains ^24^.

In recent work, we introduced geometric frustration via asymmetric patch placement on anisotropic platelets and found that partial bonding can stabilize nontrivial polymorphs with distinct structures and tunable porosity ^25^. While that study focused on slight patch displacements – just 10% of the edge length – from the particle center for a large variety of patch topologies, here we systematically investigate a single patch topology across increasing levels of asymmetry, gradually shifting the patches farther off-center. We study the impact of progressively increasing patch anisotropy on the resulting self-assembled structures. We focus in particular on the robustness of crystalline order under increasing geometric frustration, examining whether enhanced asymmetry destabilizes order or promotes new crystalline arrangements, and if patch displacement governs the competition between polymorphs.

## Methods

**Figure 1:**
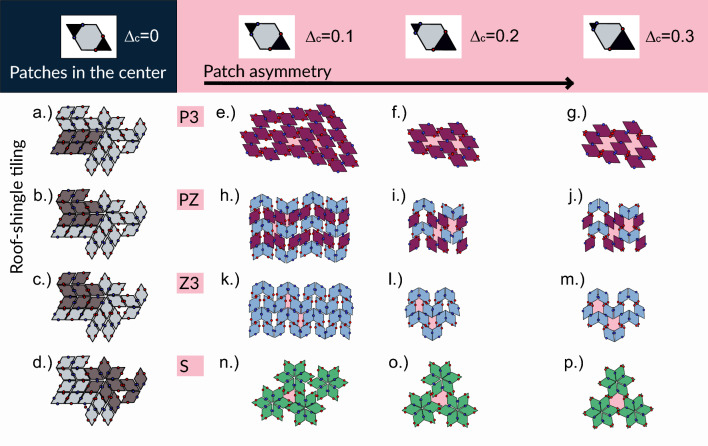
Top: particle representations on varying patch asymmetry from $\Delta _c=0$ up to $\Delta _c=0.3$. Bottom: random tiling and crystal polymorphs for different patch asymmetries $\Delta _{c}$ as observed in simulations. Panels a-d: $\Delta _{c} = 0$, roof-shingle tiling. a: tiling with parallel motif highlighted; b-c: tiling with zigzagging motif highlighted. Note that b and c are exactly the same image, highlighting that PZ and Z3 stem from the same close-packing zig-zag motif; d: tiling with flower motif highlighted. Panels e-p: crystal polymorphs, column-wise for different patch asymmetries $\Delta _c=[0.1,0.2,0.3]$. Particle colors according to the number of parallel/non parallel bonds per particle; pores highlighted in light pink. Panels e-g: parallel (P3) tiling, particles colored in burgundy display three parallel bonds. Panels h-j: zigzagging (PZ) tiling, particles in burgundy have three parallel bonds, particles in sky-blue two parallel bonds and one non parallel bond. Panels k-m: zigzagging (Z3) tiling; particle color highlights that all particles have two parallel and one non parallel bond. Panels n-p: flower-like (S) tiling; green denotes that all particles have two non parallel and one parallel bond.

**Particle model.** We consider a specific type of anisotropic colloidal platelet: a hard regular rhombus ($60^\circ$) of edge length *l=1*, functionalized with four localized interaction patches arranged, one per edge, in a selected topology, labeled dmo-s2 and introduced in Ref. ^26^. The dmo-s2 topology is characterized by two patch types, each appearing in pairs, where same-type patches are positioned to enclose the small angles of the rhombus. Patches of the same type attract each other via a short-range, square-well interaction, while patches of different types repel each other via a short-range, square-shoulder potential. The degree of patch asymmetry is tuned by the off-center displacement $\Delta _c$, defined in units of the edge length *l*. When $\Delta _c = 0$ all patches are placed at the center, while for $\Delta _c = 0.5$ patches are located at the vertices. Examples of particles with varying degrees of $\Delta _c$ are shown in the top panels of Figure 1. As this study systematically explores the effect of patch asymmetry on the self-assembly, $\Delta _c$ is varied across low (0.1), intermediate (0.2), and high (0.3) values. The total interaction between the platelet bodies of two rhombi i and j is defined via a hard-core potential:

$$U(\vec {r}_{ij}, \Omega _{i}, \Omega _{j}) = {\left\{ \begin{array}{ll} 0, & \text {if } i \text { and } j \text { do not overlap,} \\ \infty , & \text {otherwise}, \end{array}\right. }$$

where $\vec {r}_{ij}$ denotes the center-to-center displacement vector, and $\Omega _i$, $\Omega _j$ represent the particle orientations. Overlap detection is carried out using the separating axis theorem ^27^.

Patch interactions are modeled using a square-well potential:

$$W(p_{ij}) = {\left\{ \begin{array}{ll} \pm \epsilon & \quad \text {if } p_{ij} < 2r_p, \\ 0 & \quad \text {otherwise}, \end{array}\right. }$$

where $p_{ij}$ is the center-to-center distance between patches, $r_p = 0.05$ is the patch radius, and *ε = 1* sets the patch-patch interaction strength. With this choice, each patch can form at most one bond ^28^.

**Simulation details.** We employ large-scale Monte Carlo simulations in two dimensions to investigate the assembly behavior of the dmo-s2 system. The simulations include single-particle translation and rotation moves, as well as cluster moves to enhance sampling ^29,30^. For each simulation, we use *N = 1500* particles and simulate a total of *m = 16* temperatures, ranging from *T = 0.01* to *T = 0.16*, and *n = 24* packing fractions between *ϕ = 0.01* and *ϕ = 0.525*, yielding a total of 384 state points.

Each simulation is initialized from a regular lattice configuration, which is first equilibrated for $10^6$ Monte Carlo sweeps with patch interactions turned off (infinite-temperature regime). The actual assembly process is then triggered by activating the patch interactions and setting the target temperature. For statistical robustness, we perform 8 independent runs per state point, leading to 9216 simulations in total. The simulations are extended up to $2 - 2.5 \times 10^7$ Monte Carlo sweeps. For quantitative analysis, observables are averaged over the final stages of each run: we record a checkpoint every 10, 000 sweeps and aggregate the last 100 checkpoints per run, across all 8 replicas. This consistent treatment of statistics is referred to as the *standard statistical procedure* throughout the manuscript.

## Results

**Geometric frustration and polymorphism.** When all four patches are placed centrally on the particle edges (see top-left panel in Figure 1), the system self-assembles into a roof-shingle tiling, studied in detail in Ref. ^26^. In this structure, particles fully bond, i.e. form four bonds each, in two possible bond orientations – parallel and non parallel. As these two orientations occur randomly, the roof-shingle-tiling is a space filling tiling without long range positional or orientational order. In fact, even though the roof-shingle tiling forms domains of parallel, ordered rhombi, this orientational and positional order remains limited to local and intermediate length scales, as non-parallel bonding is still possible. This leads to the insertion of zig-zag and star motifs, which are all mutually commensurable, thereby preventing the emergence of global order (see Figure 1a-d for motifs of the roof-shingle tiling).

When the patches are placed only slightly off-center, i.e. for $\Delta _{c}=0.1$, particles can not bond all four patches simultaneously because it would generate particle overlaps with other neighbours, or patch misalignment due to the slight off-centered patch placements ^25^. As a result, the system becomes only partially bonded, with each particle forming, on average, three bonds. The missing fourth particle generates voids within the assembly enhancing the space of possible bonded configurations that would otherwise produce overlaps. In principle, this larger configurational space might be expected to promote disorder – an effect reported in other colloidal systems with limited patch valency between two and three ^31–34^. Surprisingly, however, we instead observed that the system organizes into partially bonded porous crystals ^25^, with at least four distinct polymorphs emerging across different state points. These findings demonstrate that even a minimal asymmetry of $\Delta _c = 0.1$ is sufficient to suppress the disordered random tiling phase and stabilize polymorphic crystalline order. We refer to the suppression of full bonding caused by the anisotropy of the bonding pattern as geometric frustration.

Here, we investigate the effect of geometric frustration on self-assembly by systematically shifting all patches off-center: one pair is moved closer to its enclosing vertex, while the other is moved away by the same amount (see top-right panel in Figure 1). To deliberately enhance geometric frustration, we go beyond minimal patch asymmetry and increase $\Delta _c$ to 0.2 and 0.3.

We begin by analyzing the self-assembled configurations across the tested values of patch asymmetry and find that crystallinity persists throughout. Across all cases, we identify four distinct polymorphs, each illustrated in Figure 1 and described in detail below. For specifics on crystal structure identification, see the Electronic Supplementary Information (ESI). It is worth noting that the bonding motifs underlying the observed structures are all already present in the roof-shingle tiling: there is a parallel bonding pattern (Figure 1a), where particles are bonded in a parallel fashion, a zigzagging bonding pattern where particle are alternately bonded parallel and non parallel (Figure 1b-c) and a star motif, where six particles are bonded into a non-parallel star pattern (Figure 1d). While these motifs are commensurate in the random tiling (Figure 1a–d), they become incommensurate as $\Delta _c>0$ giving rise to porous partially bonded crystals (Figure 1e-p). The visual inspection of simulation snapshots reveals that as the patch displacement increases beyond 0.1, the same four crystal structures assemble, but with increasing pore sizes.

The first of these polymorphs is the parallel polymorph (P3), shown in Figure 1e–g. It features three parallel bonds per particle and pores with two distinct orientations, where the pores have a non-convex s-shaped form. The P3 lattice structure reflects the parallel bonding motif seen in the close-packed tiling, and while the bonding pattern remains unchanged with increasing shift up to $\Delta _{c}= 0.3$, the pores become larger.

Next, we observe two zigzagging polymorphs: PZ and Z3 (Figures 1h–j and 1k–m, respectively). Both display three bonds per particle, and focusing the crystal columns in Figure 1h and k, they both bond in a parallel fashion. The difference arises in how these columns connect: PZ joins neighboring columns in a parallel fashion, while Z3 displays non parallel cross-column bonds. Note that in PZ this gives rise to two types of alternating bonding patterns, where one has three parallel bonds (particles colored in burgundy in Figure 1h-j and the other bonding pattern has two parallel and one non parallel bond (particles colored in sky-blue in Figure 1h-j). Z3 on the other hand only has particles with two parallel and one non parallel bonds (therefore particles only colored in sky-blue in Figure 1k-m). For PZ, the resulting pore geometries are a mix of arrow-like and s-shaped forms, while Z3 displays only arrows. For both PZ and Z3 the arrows alternate in orientation from one column to the next.

Finally, the S-polymorph (Figure 1n–p) exhibits three bonds per particle as well, but its structure consists of flower-like clusters connected by parallel bonds. This motif generates bonding patterns with one parallel and two non parallel bonds and pores shaped like truncated triangles.

**Relative polymorph abundance.** Having visually confirmed that crystallinity persists across all tested patch asymmetries, we now aim to systematically quantify the occurrence of each polymorph across different packing fractions *ϕ* and temperatures *T*. Each crystalline structure displays a distinct local bonding pattern, which we use to define an order parameter capable of distinguishing between the polymorphs. This order parameter, first introduced in Ref. ^25^ and detailed in the ESI, identifies a particle with a characteristic bonding pattern – indicating the P3, Z3, or S environments – as crystalline if it has at least one bonded neighbor with the same bonding pattern. For the PZ structure, where bonding motifs alternate, the criterion is altered such that a particle must have a neighbor with the complementary bonding pattern – i.e., a purely parallel-bonded particle must have at least one neighbor with a mixed bonding signature, and vice versa. After identifying particles as P3/PZ/Z3 or S crystalline, we calculate the size of the crystalline clusters via counting all connected P3/PZ/Z3/S particles. Clusters are only counted as crystallites if they exceed a minimum size of five crystalline particles.

**Figure 2:**
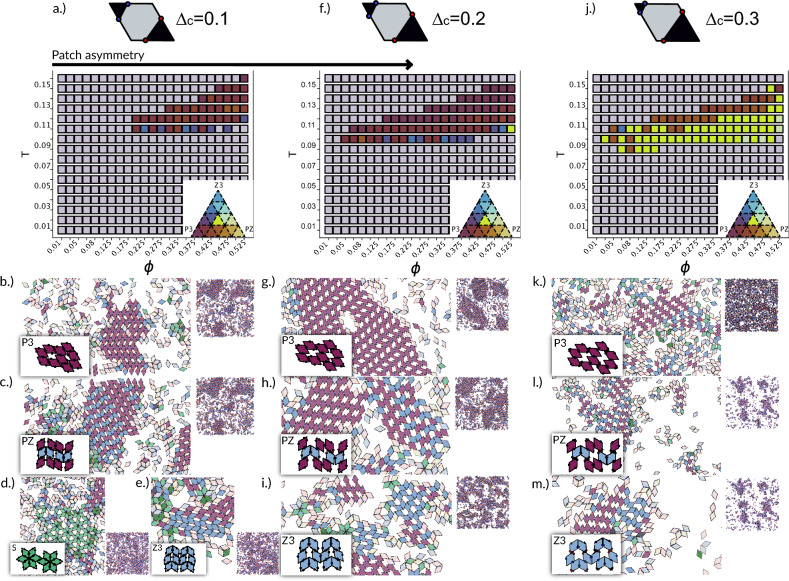
Relative prevalence of crystal polymorphs (heat maps) and simulation snapshots across different packing fractions *ϕ* and temperatures *T* or different patch asymmetries $\Delta _c$ (with the corresponding particle representations shown above each heat map). For all heatmaps grayed out areas denote state points where the overall crystallinity is below 0.05 (fraction of particles). Colors are assigned according to a barycentric color map that maps the relative crystal prevalence of the three most dominant polymorphs to a color; burgundy: more than 2/3 of crystal neighbourhoods are P3; beige: more than 2/3 are PZ; and sky-blue: more than 2/3 are Z3. If polymorphs occur close to equally often, neon yellow is taken on. We note that the crystal structure S occurs always least often for all state points and is automatically not considered in the color mapping. Configurations are taken from all checkpoints entering the standard statistical procedure. Panel a: prevalence of different crystal structures at patch asymmetry $\Delta _c=0.1$ in the *ϕ -T* plane. Panel b: snapshots of P3 crystallites for *ϕ =0.45* and *T=0.14*. Panel c: snapshots of PZ crystallites for *ϕ =0.45* and *T=0.14*. Panel d: snapshots of S crystallites for *ϕ =0.475* and *T=0.15*. Panel e: snapshots of Z3 crystallites for *ϕ =0.5* and *T=0.14*. Panel f: prevalence of different crystal structures at patch asymmetry $\Delta _c=0.2$ in the *ϕ -T* plane. Panel g: snapshots of P3 crystallites for *ϕ =0.45* and *T=0.14*. Panel h: snapshots of PZ crystallites for *ϕ =0.475* and *T=0.14*. Panel i: snapshots of Z3 crystallites for *ϕ =0.45* and *T=0.12*. Panel j: prevalence of different crystal structures at patch asymmetry $\Delta _c=0.3$ in the *ϕ -T* plane. Panel k: snapshots of P3 crystallites for *ϕ =0.5* and *T=0.14*. Panel l: snapshots of PZ crystallites for *ϕ =0.2* and *T=0.12*. Panel m: snapshots of Z3 crystallites for *ϕ =0.2* and *T=0.12*.

Figure 2 summarizes our results. For each patch asymmetry, we present crystallinity state diagrams in the *ϕ*-*T* plane alongside simulation snapshots of the observed polymorphs. The state diagrams, reported in panels a, f and j, are color-coded: regions where crystallinity exceeds *5%* are shown in color, while grey areas indicate negligible crystal formation. The color itself is mapped from a barycentric color triangle (shown in the lower right corner of each diagram), where dominant polymorphs correspond to pure hues – burgundy for P3, orange for PZ, and blue for Z3. Mixed hues indicate the coexistence of multiple polymorphs, with neon yellow marking a state where all three polymorphs appear in approximately equal proportions.

For $\Delta _c = 0.1$, the state diagram is shown in Figure 2a, with snapshots of P3, PZ, S, and Z3 crystallites in panels b–e. Crystallinity emerges for *ϕ > 0.225* within an intermediate-to-high temperature range (*T = 0.11* to 0.16). At lower packing fractions, the crystalline region is restricted to a narrow temperature window near *T = 0.11*, while at higher densities, the crystalline region spans the range *T=0.11* to *T=0.16*. P3 dominates at high temperatures (Figure 2b), gradually giving way to mixed phases with PZ at lower temperatures. At the lowest crystallization temperatures, Z3 occasionally dominates (Figure 2e), though mixed phases with P3 and PZ remain prevalent. We note that all three of these polymorphs – P3, PZ and Z3 are commensurate along one axis, allowing them to grow seamlessly together, as seen in Figure 2c for P3 and PZ. The S polymorph, shown in Figure 2d, appears only at isolated state points and never dominates. It forms distinct, isolated crystallites, as it is incommensurate with the other structures. The non-crystalline (grey) regions in the state diagrams correspond to either high-temperature liquids or low-temperature, network-like disordered phases – with the latter previously studied in detail in Ref. ^35^.

For $\Delta _c = 0.2$, P3 dominates across a much wider temperature/density range than at $\Delta _c=0.1$, which can be seen by comparing the spotty brown-burgundy colored squares of $\Delta _c=0.1$ to the more continuously burgundy colored region in $\Delta _c=0.2$. PZ becomes more prevalent only for lower temperatures, followed by a rise in Z3 at the lowest *T* values. Additionally, the crystalline region now extends to much lower packing fractions ($\phi \gtrsim 0.05$) and slightly lower temperatures (*T = 0.10*–0.15). Snapshots of P3, PZ, and Z3 crystallites for $\Delta _c = 0.2$ are shown in Figure 2g–i. Compared to the $\Delta _c = 0.1$ case, the increased patch asymmetry results in visibly larger pores within the crystal structures.

At the highest asymmetry tested, $\Delta _c = 0.3$, the extent of the crystalline region remains similar to that of $\Delta _c = 0.2$, spanning *ϕ > 0.05* and *T = 0.09*–0.15. However, the pattern of polymorph dominance changes significantly. Already at the highest temperatures, P3 and PZ occur in roughly equal proportions. Upon slight cooling, Z3 joins in, and a near-equal mixture of all three polymorphs becomes dominant across a wide range of state points, as indicated by the neon yellow color in the state diagrams. Representative snapshots of these structures are shown in Figure 2k–m. At this high asymmetry, we occasionally observe crystal rows bending significantly, though they remain connected to their surrounding particles – as clearly visible in Figure 2l. This bending results from the increased bonding flexibility introduced by extreme off-edge bonding: when particles are bonded off-edge, they can adopt a wider range of mutual orientations while still maintaining the bond. Unlike edge-to-edge configurations, off-edge bonding reduces steric hindrance during rotation, allowing particles to reorient more freely without breaking the bond (see Ref. ^36^ for a quantitative evaluation of bonding flexibility). Summarizing, for lower patch asymmetries ($\Delta _c = 0.1$ and 0.2), P3 dominates at high temperatures, with PZ and Z3 emerging sequentially as the temperature decreases. In contrast, at the highest asymmetry ($\Delta _c = 0.3$), P3, PZ, and Z3 appear in nearly equal proportions across most of the crystalline state space.

It is important to note that these observations stem from self-assembly simulations rather than free energy calculations. We speculate that the prevalence of specific polymorphs may reflect kinetic accessibility rather than thermodynamic stability. Indeed, we observe a temperature-dependent effect on the balance between polymorphs at fixed *Δ*. In contrast, no clear density-dependent trend in their relative abundance is observed, which is consistent with the fact that all three polymorphs have three bonds per particle and exhibit the same packing fraction (for fixed *Δ*). Determining the stable polymorphs across the *ϕ*–*T* plane would require free energy calculations, which are beyond the scope of this study.

**Figure 3:**
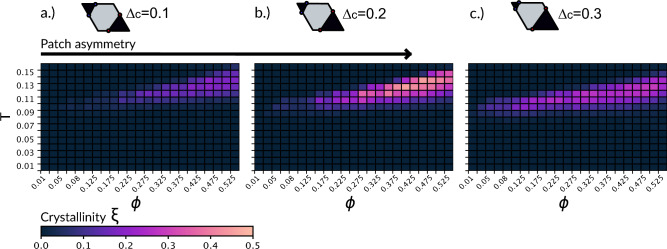
Heat map of overall crystallinity *ξ* across the whole packing fraction/temperature range of *ϕ =0.01*-0.525 and *T=0.01*-0.16 for three different patch asymmetries $\Delta _c$ (with the corresponding particle representations shown above each heat map). While *ξ* runs between 0 and 1, here the color-bar runs from the minimum observed crystallinity of 0 (black) to the maximum observed crystallinity of 0.5 (light pink). Configurations are taken from all checkpoints entering the standard statistical procedure. Panel a: *ξ* for $\Delta _c=0.1$. Panel b: *ξ* for $\Delta _c=0.2$. Panel c: *ξ* for $\Delta _c=0.3$.

**Overall crystallinity.** As illustrated by the snapshots in Figure 2, none of the assemblies reach full crystallization across the range of patch asymmetries. Unbonded particles remain in the system, and many bonded particles are part of unordered aggregates or located at the surfaces of crystallites without exhibiting local crystalline order. To quantify overall crystallinity, we calculate *ξ*, the fraction of bonded particles that are part of any of the identified polymorphic crystallites. The results are shown in the heat maps in Figure 3a-c, where the color map runs from black (close to zero, low crystallinity) to light pink (close to 0.5, highest found crystallinity, half of the bonded particles are crystalline). The heat map covers all state points from packing fractions *ϕ =0.01* to *ϕ =0.525* and temperatures from *T=0.01* to *T=0.16*. For the lowest patch asymmetry, $\Delta _{c} = 0.1$, the overall crystallinity remains small – i.e. *ξ* between 0.1 and 0.2 – with the highest values observed at packing fractions *ϕ > 0.375* and temperatures *T ≈ 0.13*–0.14. For $\Delta _{c} = 0.2$, we observe a significant increase in crystallinity: values reach up to 0.5 for *ϕ > 0.275* and *T ≈ 0.12*–0.14. This intermediate patch asymmetry appears to present a “sweet spot” for crystallinity, as for the highest asymmetry, $\Delta _{c} = 0.3$, crystallinity decreases again. Although still slightly higher than at $\Delta _{c} = 0.1$, crystallinity remains well below the peak values observed at $\Delta _{c} = 0.2$, with maximum crystallinity around 0.25 for packing fractions *ϕ > 0.125* and *T ≈ 0.12*–0.14. This non-monotonic behavior in crystallinity is also reflected in the simulation snapshots in Figure 2: the size of P3 crystallites varies noticeably with patch asymmetry. For $\Delta _{c} = 0.2$ (panel g), the crystallites are visibly larger than those observed for both $\Delta _{c} = 0.1$ (panel b) and $\Delta _{c} = 0.3$ (panel k).

These observations raise two central questions: why does overall crystallinity remain relatively low across all patch asymmetries? And why does a pronounced “sweet spot” in crystallinity emerge at $\Delta _c = 0.2$? Regarding the first question, we observe that while the initial formation of small crystallites occurs relatively quickly, their subsequent growth proceeds only slowly. The slow crystal growth may result from the competition between multiple polymorphs close in free energy. In Ref. ^25^, we explored significantly longer simulation times at $\Delta _c = 0.1$ for this and other geometrically frustrated patch topologies and observed that crystallites do continue to grow, albeit very slowly: even after $3\times 10^7$ Monte Carlo sweeps, most assemblies remained only partially crystallized. From the snapshots we estimate that there are still enough particles in solution for all crystallizing densities, such that we speculate that it does not seem to be particle depletion alone that hinders the growth. Instead, we surmise that it may be the polymorphic competition at the free energy level that leads to frequent re-orderings and thus impedes large-scale ordering.

Similarly the enhanced crystallinity observed at intermediate patch asymmetry ($\Delta _c = 0.2$) may be due a change in the relative free energies: we speculate that the P3 polymorph becomes comparatively more stable than the others, reducing polymorphic competition and enabling more extensive crystallization. This assumption is supported by the observation that the prevalence of P3 is largest and most extended in *ϕ -T* for $\Delta _c=0.2$. While in both cases, free energy calculations would be required to determine the underlying polymorph stability with certainty, kinetic factors such as nucleation barriers, bonding flexibility, or defect healing rates may also play a role, suggesting that both energetic and dynamical considerations should be taken into account in order to fully understand these phenomena.

**Porosity of partially bonded crystals.** In our previous work ^26^, as well as in experimental studies ^37–39^, it was shown that off-edge bonding – where particles are laterally offset rather than aligned edge-to-edge – can lead to fully bonded porous crystal structures^40^. Building on this, we demonstrated that porosity in fully bonded Kagome and rhombic crystals can be tuned by adjusting the off-center placement of patches in a particular patch topology (referred to as dma-as2 in previous works, see Ref. ^26^). In the present study, the partially bonded polymorphs are also porous. However, in these partially bonded polymorphs, porosity arises not only from off-edge bonding but also from the voids created by leaving one of the four patches unbonded. We thus explore whether fully or partially bonded structures produce larger pores and the degree to which porosity can be controlled in the partially bonded polymorphs.

**Figure 4:**
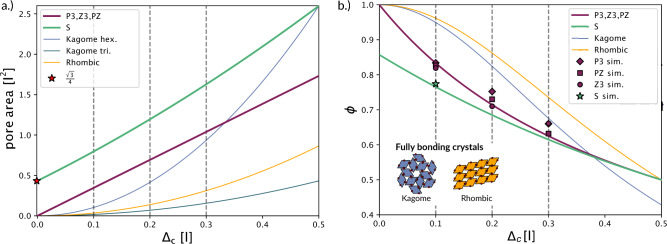
Panel a: pore area (size) of all occurring pore shapes in partially (P3, PZ, Z3, S) and fully (Kagome and rhombic) bonded assemblies as function of patch asymmetry $\Delta _c$ and in units of squared rhombi edge length ($l^2$).Pore area of P3, PZ and Z3 pores (burgundy), S-pores (green), hexagonal Kagome pores (blue), triangular Kagome pores (turquoise) and rhombic pores (orange). Red star denotes the area of triangular S-pores at $\Delta _c=0$. Vertical dotted lines indicate $\Delta _c$-values of the simulation runs. Panel b: packing fraction of partially and fully bonded monolayers as function of patch asymmetry $\Delta _c$. Packing fraction of P3, Z3 and PZ lattices (burgundy), S-lattice (green), Kagome lattice (blue) and rhombic lattice (orange). Vertical dotted lines indicate $\Delta _c$-values of the simulation runs. Inset: fully bonded Kagome (blue) and rhombic (orange) lattices.

Figure 4 compares the pore areas of all observed partially bonded crystals across a range of patch asymmetries. Moreover we contrast them to the pore areas of previously observed fully bonded crystals, namely the Kagome and the rhombic lattice ^26^ . Pore areas are calculated trigonometrically and expressed in units of $l^2$, the squared edge length of the rhombus (see ESI for details). Considering the partially bonded polymorphs first, we find that despite their distinct geometries – s-shaped (P3), arrow-shaped (Z3), and alternating (PZ) – the P3, PZ, and Z3 polymorphs exhibit identical pore areas for a given $\Delta _c$ (burgundy line in Figure 4a). Starting from the close-packed monolayers at $\Delta _c = 0$, the pore area increases nearly linearly, reaching $\sim 1.7l^2$ at $\Delta _c = 0.5$, roughly twice the area of a single rhombus, that has an area of $a _r = l^2*\sin {\pi /3} \approx 0.866l^2$. The S-crystal (green line), which is not close-packed even at $\Delta _c = 0$ (red star), features triangular pores that grow into large truncated triangles, reaching an area of $\sim 2.5 l^2$ – about three times the particle area – making it the largest pore across all $\Delta _c$. Among fully bonded crystals, the Kagome lattice (blue line, see lattice in the inset of Figure 4b) exhibits triangular and hexagonal pores that emerge with increasing $\Delta _c$. The hexagonal pore grows faster and eventually exceeds the pore area of P3/PZ/Z3 at $\Delta _c \approx 0.32$, approaching S-crystal pore area near $\Delta _c = 0.5$. In contrast, the triangular Kagome pore and the rhombic crystal pore (red line, see lattice in the inset of Figure 4b) remain smaller across all patch asymmetries.

More than the pore size alone, it is the overall packing fraction – i.e., the fraction of space occupied by particles – that indicates the overall porosity of the monolayer. It depends not only on the size of individual pores but also on their frequency and distribution within the lattice. To this end, we calculate the packing fraction of the full monolayers both analytically and from simulation snapshots. The analytical calculation is based on the trigonometric determination of pore area, combined with counting the number of pores and rhombi in each unit cell to compute the volume fraction occupied by particles (see ESI for details). In the theoretical calculation the patches are point-like, that means orientational fluctuations due to the patch extent are not taken into account. We also provide an empirical estimate of the packing fraction, that uses a number of simulation snapshots of the crystallites, where colored pixels – corresponding to rhombus particles – are identified and counted. From here, the ratio of colored-to-total pixels is calculated, which is equivalent to the packing fraction (details are provided in the ESI).

For all structures, the packing fraction decreases monotonically with increasing $\Delta _c$. All polymorphs except the S-crystal have *ϕ = 1* at $\Delta _c = 0$, while the S-crystal exhibits a lower packing fraction due to the presence of triangular pores even when the patches are centrally placed. For $\Delta _c = 0.1$–0.3, partially bonded structures exhibit lower packing fractions than fully bonded ones. Around $\Delta _c \approx 0.37$, the S-crystal and P3/PZ/Z3 converge in packing fraction. Interestingly, at roughly the same $\Delta _c\approx 0.37$ the reference Kagome structure with its hexagonal and triangular pores becomes more porous than the partially bonded crystals. The reference rhombic crystal consistently shows the highest packing fraction/lowest porosity across the entire range of $\Delta _c$. In summary, partially bonded crystals form larger pores and exhibit lower packing fractions than fully bonded structures at the simulated patch asymmetries. For higher patch asymmetries, the fully bonded Kagome lattice exceeds in porosity, while the rhombic lattice remains the densest packing throughout all patch asymmetries. We conclude by noting that, as much as for full-bonding, pores generated by partial bonding can be fine tuned with the patch asymmetry, and thus may be a valuable design feature of engineering materials with controllable porosity.

## Conclusions

Patchy colloids are typically designed to favor full bonding within a target crystal structure. This strategy has enabled the directed self-assembly of a wide range of ordered structures. Nonetheless, relaxing the constraint of full bonding opens alternative pathways to crystallinity. In earlier work, we demonstrated that deliberately introducing geometric frustration through a combination of asymmetric patch placement and anisotropic particle shape, we can tilt the system away from a close-packing disordered tiling towards a rich set of porous ordered structures.

Here, we systematically increase the degree of patch asymmetry to explore its impact on self-assembly.

We find that patch placement selectively stabilizes different polymorphs, modulates porosity, and controls crystal yield. When interaction patches are positioned centrally on the particle edges, the system forms a roof-shingle tiling, a random tiling with no long-range order. However, shifting patches off-center unlocks the emergence of four distinct, ordered polymorphs, exhibiting varying degrees of porosity. As patches are displaced further from the edge centers, the resulting crystal structures become increasingly porous. This effect stems from the fact that off-center placement limits the number of bonds each particle can form, leaving more space between bonded units. Partially bonded structures, where some patches remain unbonded, are thus particularly notable for their enhanced porosity compared to fully bonded crystals found in other patch topologies. Interestingly, crystallinity does not monotonically increase or decrease with patch asymmetry. Instead, there is an optimal intermediate offset where the system achieves the highest degree of crystallinity. At this point, frustration limits full bonding and prevents disordered tilings, but not to the extent that it inhibits the formation of ordered patterns. This indicates that a balance between bonding constraints and structural flexibility is crucial for high-quality crystal formation. Furthermore, the identity of the dominant polymorph varies sensitively with patch placement, indicating that patch geometry acts as an effective control parameter for selecting specific lattice types. Taken together, these results show that geometric frustration can be used not only to generate porous crystals, but also to steer polymorph selection and optimize crystal yield.

Our results can be related to phenomena well known in organic and supramolecular chemistry, where partially satisfied hydrogen or halogen bonds give rise to complex polymorphic landscapes ^41–45^. These polymorphs can differ not only in packing but also in molecular conformation ^46,47^, leading to materials with distinct porosities, stabilities, and physical properties ^47,48^. Despite significant progress in understanding polymorphism, fundamental questions remain open – such as why some compounds exhibit multiple forms, which polymorphs are experimentally accessible under given conditions, and how to predict structure-property relationships ^49^. The parallelism with our functionalized platelets suggests that anisotropic patchy colloids – where geometry and patch positioning play a key role in determining the accessible crystal structures – may serve as simplified model systems for investigating polymorphism in chemical compounds, that are usually non-spherical in shape as well and possess functional groups, that may be coarsed-grained to patches ^26,50,51^. Conversely, established concepts from molecular crystallography can inform the design of colloidal materials with targeted structural and functional properties, including porosity, responsiveness, and reconfigurability.

## Supplementary Information

Supplementary Information.

## Data Availability

Software used for Monte-Carlo simulation and data analysis is available here: https://github.com/OljaOctagon/Colloidal-Assembler/. Simulation data is available upon request via carina.karner@tuwien.ac.at
