# Supplementary material for: Effect of asymmetry on frustrated crystallization
Source: Sci Rep. 2026 Jun 4;16:25603. doi: 10.1038/s41598-026-53474-8 (PMC13478378; doi:10.1038/s41598-026-53474-8)
Supplement: Supplementary file 1 [file 41598_2026_53474_MOESM1_ESM.pdf]

Supporting Information of "Effect of asymmetry on frustrated crystallization"

Carina Karner*

Institut für Theoretische Physik, TU Wien,
Wiedner Hauptstraße 8-10, A-1040 Wien, Austria

Emanuela Bianchi†

Institut für Theoretische Physik, TU Wien,
Wiedner Hauptstraße 8-10, A-1040 Wien,
Austria and CNR-ISC, Uos Sapienza,
Piazzale A. Moro 2, 00185 Roma, Italy

(Dated: April 17, 2026)

I. CRYSTALLINE STRUCTURE IDENTIFICATION IN THE DMO-S2 SYSTEM

Generally, a particle can be considered as crystalline, if the neighourhood around the particle displays crystalline order – i.e the relative positions and orientations of the particle's neighours are such that, the whole neighbourhood adheres to a specific crystal symmetry. Ordinarily, one would therefore rely on symmetry detecting positional order parameters such as the Steinhardt bond order parameters, that take into account distance vectors of the nearest neighbours for crystal structure detection [**?**]. In our system, however, these metrics are not suitable. The crystalline structures formed here – due to the shape anisotropy – depend not only on particle positions but also on particle orientations, and – due to the bonding anisotropy – the directionality of patch–patch interactions. Similarly, global structure identification methods, such as radial distribution function or structure factor, are insufficient because of the presence of competing polymorphs. This means that we would not be able to assign emerging crystal peaks to a specific crystal structure. Instead, we base our identification scheme on local bonding patterns, which capture both positional and orientational constraints and are sufficiently distinct between the crystal types observed in this system.

Local bonding patterns. A regular patchy rhombus can bond to another rhombus either via a parallel (p) bond or a non parallel (np) bond. The dmo-s2 patch topology has four small patches, where one patch bonds one neighbourhing particle, hence every particle can bond to up to four particles. Based on these observations, we define the bonding pattern of a particle as the number of parallel (p) and non parallel (np) bonds. By recording to how many parallel and non parallel particles a particle is bonded to we can algorithmically detect all occurring bonding patterns. As naming scheme we chose A-np-B-p, where A denotes the number of non parallel bonds and B denotes the number of parallel bonds. If one – either A or B – are zero the name is shortened to A-np or B-p. In Figure S1 particles are shown as colored according to their bonding patterns (see the legend in Figure S1d). Already from visual inspection we can observe four dominant bonding patterns of the four types of ordered domains: the P3-structure, built from particles with three parallel bonds (3-p) in burgundy, the Z3-structure based on a 1-non-parallel–2-parallel (1-np-2-p) bonding configuration in light-blue, the PZ-structure, which combines particles following a 3-p bonding pattern with

* carina.karner@tuwien.ac.at
† emanuela.bianchi@tuwien.ac.at

particles following a 1-np-2-p bonding pattern in an alternating fashion (in burgundy and light-blue), and finally, the S structure, which displays a 2-np-1-p bonding pattern shown in sea-green. These observations indicate that the dominant crystal types can be distinguished based solely on their local bonding patterns. This bonding-pattern identification therefore forms the basis of our crystal-structure detection.

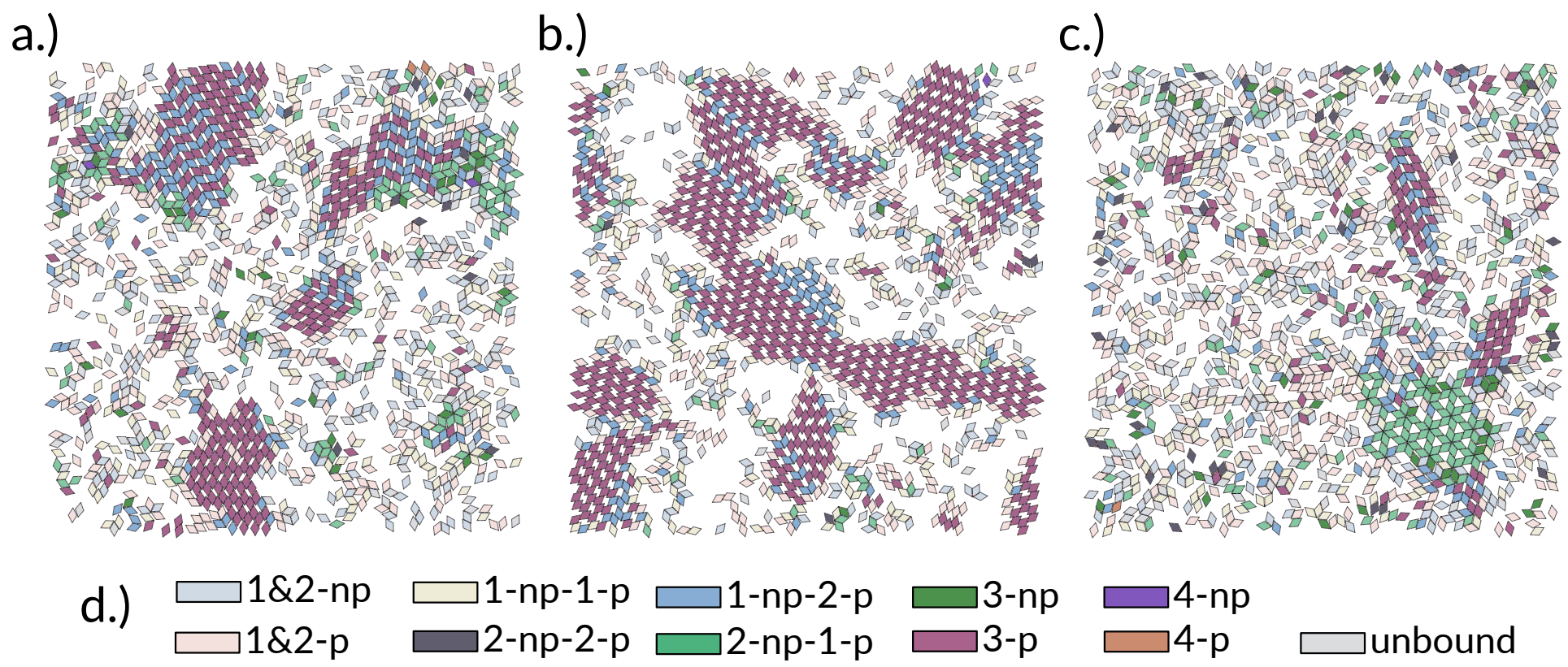

Figure S1. **Bonded neighbourhoods in sample snapshots for some of the investigated systems.** (a) $\Delta_c = 0.1$, $\phi = 0.45$, $T = 0.14$, (b) $\Delta_c = 0.1$, $\phi = 0.425$, $T = 0.13$) and (c) $\Delta_c = 0.1$, $\phi = 0.475$, $T = 0.15$. Generally label abbreviations follow the scheme A-np-B-p, where A is the number of non parallel bonds and b the number of parallel bonds. For example, 2-np-2-p denotes a particle with two non-parallel and two parallel bonds. Exceptions to this scheme are 1&2-np and 1&2-p, that indicate particles with either one or two non parallel (np) or one or two parallel (p) bonds. The full legend is given in (d).

Pattern-based classification of crystal structures. To robustly identify crystalline particles, we implement a rule-based classification scheme. Each crystal type is defined by two criteria: (i) a target pattern, which describes the ideal local bonding configuration of a particle and its immediate neighbors, and (ii) a threshold condition, which allows for local deviations or defects.

Therefore, a particle is classified as part of a P3-domain if it is 3-np bonded and at least one of its bonded neighbors is also 3-np bonded. The same logic applies to Z3-domains, where the particle and at least one neighbor must both exhibit the 1-np-2-p pattern. For the PZ-structure, which consists of alternating bonding motifs, we classify a particle as PZ-crystalline if it exhibits either a 3-p or a 1-np-2-p pattern and has at least one neighbor

exhibiting the complementary motif. This captures both subtypes required to stabilize the PZ-structure. For the S structure, we require the particle itself to be 2-np-1-p bonded and have at least one neighbour of the same bonding pattern.

Cluster validation and filtering. Since local bonding patterns can occasionally occur by chance in the disordered phase, we include an additional filtering step to distinguish genuine crystallites from spurious configurations. A particle is only marked as crystalline if it is part of a continuous cluster containing at least five other crystalline particles of the same type. This filtering eliminates small, transient aggregates that do not represent persistent crystalline order.

Detection results. Figure S2 presents snapshots at different state points, with crystalline particles highlighted according to the classification described above. Panel (a) shows a system dominated by P3 motifs, forming extended domains, panel (b) shows crystal domains in the PZ-structure, panel (c) displays dispersed Z3-like regions, which are less well-structured and appear mostly in isolated lanes, while the final panel (d) shows a rather spherical S-crystallite. The insets at the bottom of the panels repeat the target and threshold rules outlined above.

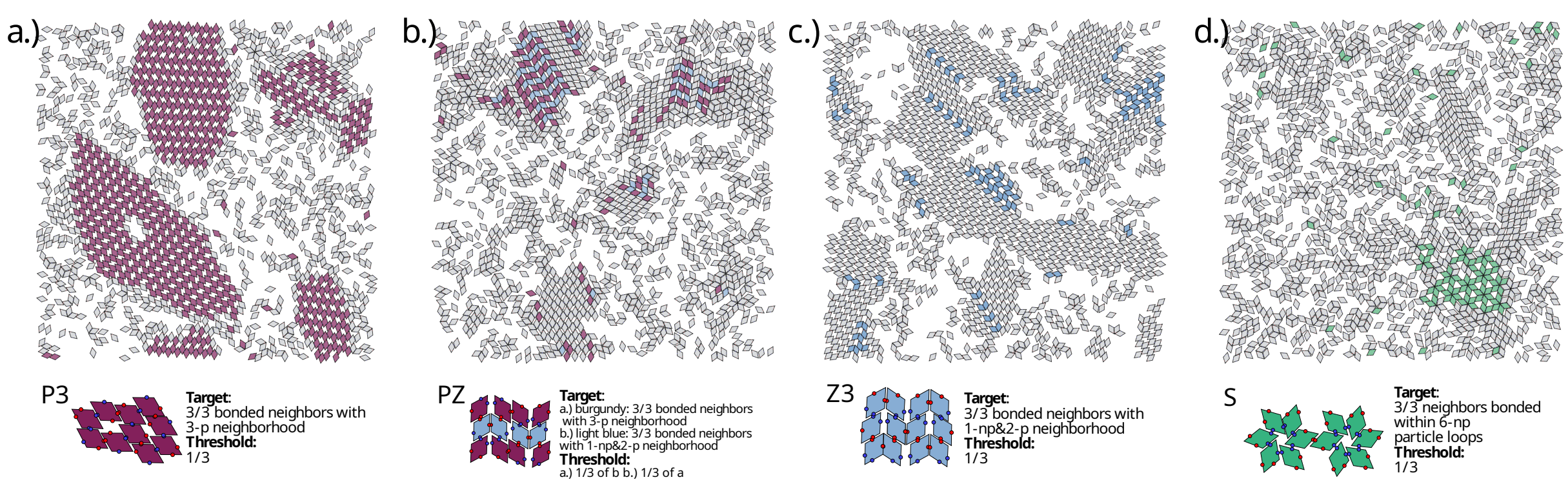

Figure S2. **Crystal structure detection for dmo-s2.** (a) P3 at at $\Delta_c = 0.2$, $\phi = 0.45$, $T = 0.14$, (b) PZ at $\Delta_c = 0.1$, $\phi = 0.45$, $T = 0.14$, (c) Z3 at $\Delta_c = 0.1$, $\phi = 0.425$, $T = 0.13$ and (d) S at $\Delta_c = 0.1$, $\phi = 0.475$, $T = 0.15$. Insets: target and threshold number of crystalline neighbours for crystal structure detection.

II. EVALUATION OF POROSITY AND PACKING FRACTION

Pore area calculation in the sticky limit. We calculate the pore areas of the resulting crystal structures using trigonometry. For this calculation we neglect the physical extent of the patches and assume that the patches are point-like, making this calculation a lower bound for the pore area. In the literature the limit of infinitely small patch is known as sticky limit. Note that with a patch radius of 0.05 (in units of the rhombus edge length) our patches are very small, rendering this calculation a reasonable approximation. The result of this calculation can be viewed in Figure 4a of the main text.

For the P3-crystal, the pore area A_{P3} as a function of the patch asymmetry Δ_c is given as

$$A_{P3} = \sqrt{3} \cdot l \cdot \Delta_c, \tag{1}$$

where l is the edge length of the rhombus. This relation is derived from the realization that every pore consists of two equal parallelograms with edge lengths l and Δ_c and a small angle of $\pi/3$.

Turning to the Z3 and PZ-pores, we find that their pore areas are, in fact, equal to A_{P3}. Although their pore shapes differ, both the Z3 and PZ-pores also consist of two parallelograms with the same edge lengths and angle as P3. While for P3, the two parallelograms are joined together to form a chair shape, for Z3 the parallelograms are joined to form an arrow shape. For PZ we observe an alteration of the chair and arrow shape. Therefore, we conclude that

$$A_{P3} = A_{Z3} = A_{PZ}. \tag{2}$$

S-crystal pores posses the geometry of (truncated) triangles given by the formula

$$A_S = \frac{\sqrt{3}}{4} \cdot l^2 \cdot ((1 + 2\Delta_c)^2 - 3\Delta_c^2). \tag{3}$$

In contrast to the P3/Z3/PZ-pores that vanish for the central patch position, the pores of the S crystals are equilateral triangles with the area $\sqrt{3}/4 \cdot l^2$ at $\Delta_c = 0$. As the patches move off-center the area of the triangle pore grows with a side length of $1 + 2\Delta_c$, while at the same time getting truncated at all three edges by smaller triangles with edge length Δ_c.

Packing fraction in the sticky limit. As in the case of with the pore area, trigonometry also allows us to calculate the exact packing fraction in the sticky limit. For this

calculation, we sum up the number of particles per number of pores and divide this number by the total area occupied by particles and pores for each Δ_c. The resulting packing fractions are displayed in Figure 4b in the main text. For the P3,Z3 and PZ-crystals, we find that a single pore is surrounded by 6 particles, while each particle is contact with three pores. Therefore the total area occupied by particles – i.e the packing fraction as function of the patch asymmetry Δ_c – is given by

$$\phi_{P3/PZ/Z3}(\Delta_c) = \frac{2A_r}{2A_r + A_{P3/PZ/Z3}(\Delta_c)} \tag{4}$$

where A_r is the area of a regular rhombus with edge length 1 and $A_{P3/PZ/Z3}(\Delta_c)$ is the pore area defined in Equation 1.

For the S-crystal a single pore is surrounded by six particles, and every particle is contact with two pores. Therefore, the packing fraction for the S-crystal as function of the patch asymmetry Δ is given by

$$\phi_S(\Delta_c) = \frac{3A_r}{3A_r + A_S(\Delta_c)}, \tag{5}$$

where $A_S(\Delta_c)$ is given by Equation 3.

Packing fraction at finite patch size via image analysis. Because the patches interact via a square-well potential, they do not contribute to the excluded volume. Consequently, calculating the packing fraction of each crystallite at the finite patch radius used in the simulations ($r_p = 0.05$) is not straightforward.

To estimate the packing fraction at finite patch size, we instead use image analysis. For each crystal type and patch asymmetry Δ_c, we obtain five zoomed-in screenshots of the simulation snapshots that display the bulk part of a single crystallite without the phase boundaries, with rhombi shown in color and pores in white. The packing fraction for each snapshot is determined by counting the number of colored pixels relative to the total number of pixels. The final packing fraction is then computed by averaging the packing fractions of all five snapshots. These values are reported in Figure 4b of the main text.
